# Influence of subcutaneous adipose tissue index on prognosis in cirrhotic patients following endoscopic therapy: a retrospective cohort study

**DOI:** 10.1186/s12944-023-01996-9

**Published:** 2024-01-08

**Authors:** Yongshuai Liu, Huijun Chang, Yunqing Zeng, Jinhou Li, Yueyue Li, Yong Chen, Tao Zhou, Yanjing Gao

**Affiliations:** 1 Department of Gastroenterology, Qilu Hospital of Shandong University, 107 West Wen Hua Road, Jinan, 250012 Shandong China; 2 Department of Gastroenterology, Taian City Central Hospital, Taian, Shandong China; 3 Department of Gastroenterology, Shandong Provincial Hospital, Shandong First Medical University, Jinan, Shandong China; 4 Department of Geriatric Medicine, Qilu Hospital of Shandong University, Jinan, Shandong China

## Abstract

**Background:**

The relation of adipose tissue depletion with prognostic outcome of variceal bleeding among cirrhotic patients is still inconclusive. The present work explored whether adipose tissue, which was measured based on computed tomography (CT), was valuable for analyzing rebleeding and mortality among patients with variceal bleeding who had undergone endoscopic therapy.

**Methods:**

The study encompassed cirrhotic patients who underwent endoscopic therapy to prevent variceal rebleeding between January 2016 and October 2022. The L3-level CT images were obtained. Besides, impacts of subcutaneous adipose tissue index (SATI), visceral adipose tissue index (VATI), as well as total adipose tissue index (TATI) on rebleeding and mortality among cirrhotic patients following endoscopic therapy were examined.

**Results:**

In this work, our median follow-up period was 31 months. Among those adipose tissue indexes, only SATI exhibited an independent relation to higher rebleeding (HR 0.981, 95% CI, 0.971–0.991, *p* < 0.001) and mortality (HR 0.965, 95% CI, 0.944–0.986, *p* = 0.001) risks. Upon multivariate Cox regression, low SATI (male < 30.15 cm^2^/m^2^, female < 39.82 cm^2^/m^2^) was independently linked to higher rebleeding risk (HR 2.511, 95% CI, 1.604–3.932, *p* < 0.001) and increased mortality risk (HR 3.422, 95% CI, 1.489–7.864, *p* = 0.004) after adjusting for other predictors. Furthermore, subgroups were created based on using nonselective β-blockers (NSBBs), demonstrating that quantitatively assessing SATI exerts a vital role in evaluating rebleeding incidence in patients with or without NSBB therapy.

**Conclusion:**

This study underscores the potential of quantifying SATI as a means for achieving a more accurate risk classification for individual patients and identifying patients that can gain more benefits from nutritional intervention.

## Introduction

Esophagogastric variceal bleeding (EGVB) constitutes a primary factor contributing to mortality in cirrhotic patients afflicted with portal hypertension [1]. Individuals recovering from their initial acute variceal bleeding episode face elevated risks of rebleeding and mortality, estimated at approximately 60% and 33% within one to two years, respectively [2]. The recommended approach for preventing rebleeding and extending survival time is a combination of endoscopic treatment and nonselective β-blockers (NSBBs), often referred to as the “standard therapy“ [3]. However, a noteworthy proportion of cirrhotic patients, ranging from 14 to 23%, still experience rebleeding despite the implementation of the “standard therapy,” and the rebleeding rates remain between 19% and 47% with endoscopic therapy alone [4]. Given these adverse outcomes, it is imperative to elucidate the underlying risk factors for secondary prophylaxis and the identification of high-risk rebleeding and mortality among patients.

Adipose tissue is primarily categorized as subcutaneous or visceral adipose tissue (SAT or VAT). These adipose tissue types have crucial effects on regulating appetite, inflammation, fat and glucose metabolism, angiogenesis, as well as insulin sensitivity through the secretion of various adipokines [5]. More and more studies are conducted to analyze the substantial impact of adipose tissue irregularities, which may be linked to elevated portal pressure, ultimately resulting in hepatic decompensation and fatality [6–8]. For instance, a decrease in the subcutaneous adipose tissue index (SATI) may be frequently related to adverse outcomes and higher mortality rates among cirrhotic patients, as well as those with liver cancer [9, 10]. Additionally, some research suggests that the higher VAT volume is strongly related to liver fibrosis progression, with an unfavorable prognostic outlook among non-alcoholic fatty liver disease (NAFLD) patients [11, 12].

Given the potential link between adipose tissue type and the prognosis of liver cirrhosis patients, it is hypothesized that adipose tissue irregularities can serve as prognostic indicators for cirrhosis patients following endoscopic therapy. To date, there have been limited investigations directly addressing the relation of adipose tissue distribution with prognostic outcome in cirrhotic patients experiencing variceal bleeding and undergoing endoscopic therapy. The present work focused on exploring whether adipose tissue, as measured by CT, was valuable for predicting variceal rebleeding and mortality in liver cirrhosis patients following endoscopic therapy.

## Patients and methods

### Objects of study

The current retrospective assessment recruited liver cirrhosis patients who underwent secondary prevention of variceal rebleeding at Qilu Hospital of Shandong University, Taian City Central Hospital, and Shandong Provincial Hospital between January 2016 and October 2022. Patients below were excluded: (1) those with the age of < 18 or > 80 years; (2) individuals previously undergoing endoscopic treatment, splenectomy, liver transplantation, or transjugular intrahepatic portosystemic shunt (TIPS); (3) those with the diagnosis of hepatocellular carcinoma or other extrahepatic cancers; (4) patients who did not undergo abdominal CT before treatment; (5) individuals lost to follow-up within three months. Our study protocols followed the Declaration of Helsinki and obtained approval from Ethics Committee at each institution. Due to the retrospective nature, no informed consents were required.

### Clinical and laboratory data

Patient baseline characteristics, including gender, age, body mass index (BMI), primary cirrhosis etiology, and comorbidities, were collected at the time of the initial endoscopic therapy. We obtained routine laboratory parameters such as hemoglobin (Hb) level, platelet count, total bilirubin, serum creatinine, serum albumin, prothrombin time (PT), international normalized ratio (INR), and D-dimer upon admission. Additionally, presence or absence of ascites was recorded. The study also documented Child-Pugh and Model for End-Stage Liver Disease (MELD) scores. Two physicians participated in data collection, which was subsequently verified by a third researcher.

### Endoscopic and NSBBs treatment

Endoscopic variceal ligation (EVL) was performed under intravenous anesthesia using commercially available multiband devices. Variceal ligation extended from the cardia to the oral side, and band number was determined according to varices with red spots and bleeding signs, as assessed by the operator. When necessary, patients with gastroesophageal varices received endoscopic tissue adhesives such as N-butyl cyanoacrylate. EVL was conducted at intervals of 28 to 42 days until variceal eradication was achieved. Furthermore, NSBBs, specifically carvedilol or propranolol, were administered at standard doses to eligible patients after contraindications were ruled out.

### Adipose tissue measurement

In this study, SliceOmatic software (version 5.0; Tomovision, Magog, Canada) was adopted for analyzing abdominal CT images obtained at the third lumbar vertebra level (L3). Standard Hounsfield Unit (HU) thresholds, including − 190 to -30 HU and − 150 to -50 HU for subcutaneous and visceral adipose tissue (SAT and VAT), respectively, were utilized to determine adipose tissue cross-sectional areas [13]. SAT was quantified within abdominal wall, while adipose tissue located between skin boundary and the outer abdominal wall was categorized as SAT. Total adipose tissue was calculated as combined cross-sectional area of SAT and VAT. These areas were standardized through the division of each area by square of patient’s height in meters, yielding the SATI, visceral adipose tissue index (VATI), as well as total adipose tissue index (TATI).

### Follow-up and study endpoints

Patients were continuously monitored through telephone interviews, outpatient visits, and/or the examination of medical records until they underwent liver transplantation, experienced mortality, or the study concluded. Patients who received liver transplants were considered alive, and their follow-up was monitored during the transplantation process. The final follow-up date was in May 2023. Our primary and secondary endpoints included rebleeding after initial endoscopic treatment and all-cause mortality, respectively. Rebleeding was identified during follow-up through any occurrence of hematemesis and/or melena and subsequently confirmed via endoscopy.

### Statistical analysis

Data analysis was completed with SPSS 26.0. Quantitative results were represented by means ± standard deviation (SD) or as medians and interquartile ranges (IQR) and analyzed with Student’s t-tests or Mann-Whitney U-tests. Categorical data were represented by counts and percentages and analyzed with the use of Chi-square test. Initially, the predictive potential of continuous adipose tissue characteristics for rebleeding was evaluated. Subsequently, the optimum threshold of continuous adipose tissue variables for predicting rebleeding was detected with X-tile software (version 3.6.1). The roles of adipose tissue characteristics in mortality and rebleeding after endoscopic therapy were analyzed based on Kaplan-Meier curves with log-rank tests. All factors independently predicting rebleeding and mortality were identified through univariable and multivariable Cox regression. We incorporated factors of p < 0.1 from univariate Cox regression into multivariate regression with the forward LR (forward stepwise regression based on maximum likelihood estimation) method to evaluate hazard ratios (HRs). A sub-analysis based on the use of NSBBs was performed, and *p* < 0.05 (two-sided) suggested statistical significance.

## Results

### Patient selection

**Fig. 1:**
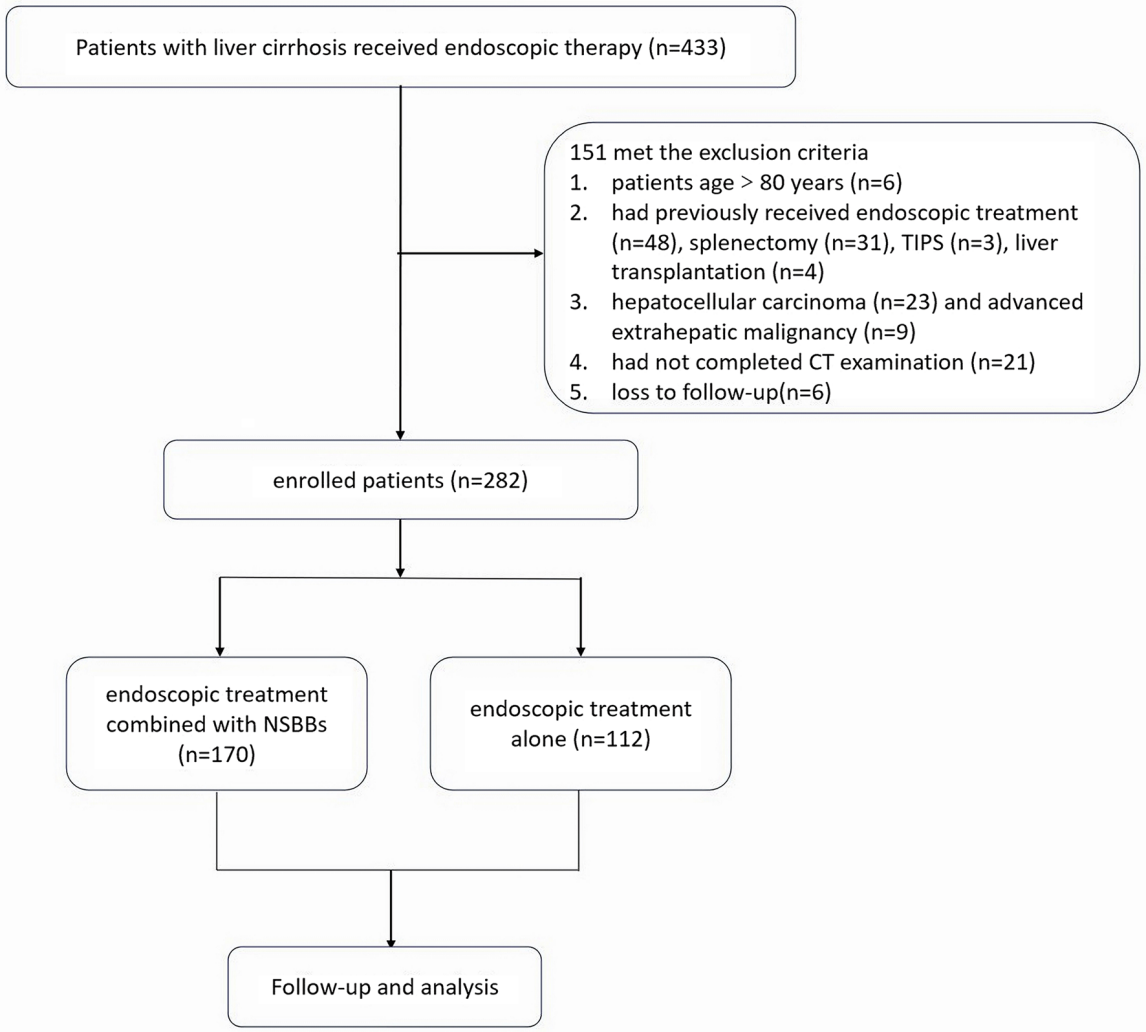
Flowchart. Retrospective selection process of patients

Between January 2016 and October 2022, totally 433 liver cirrhosis patients underwent endoscopic therapy to prevent rebleeding from gastroesophageal varices. Following the exclusion of 151 patients, a final cohort of 282 patients was involved in the analysis. Figure 1 displays the study selection procedure.

### Basic patient features

Table 1 exhibits basic features of the 282 patients. These patients had an average age of 55 years, and there were men (n = 167, 59.2%) than women. Hepatitis B was a major pathogenic factor of liver cirrhosis (n = 140, 49.6%). Additionally, the average Child-Pugh and MELD scores were 6.57 ± 1.49 and 10.09 ± 2.39, respectively. Endoscopic therapy combined with NSBBs was administered to 170 (60.3%) patients. The mean BMI for all patients was 23.65 ± 3.09 kg/m^2^. Female patients exhibited higher SATI (53.77 ± 25.08 vs. 36.85 ± 22.22 cm^2^/m^2^) and TATI (91.41 ± 39.01 vs. 74.39 ± 41.29 cm^2^/m^2^) compared to male patients (*p* < 0.001 for each). VATI did not differ significantly between males and females.

**Table 1:** Patient clinical characteristics by sex at the time of adipose tissue assessment

Characteristics	All patients (n = 282)	Male (n = 167)	Female (n = 115)	*p*-value
Age (years)	54.70 ± 10.77	51.19 ± 10.37	59.8 ± 9.21	< 0.001
**Etiologic cause, n (%)**				
Hepatitis B	140(49.6%)	101(60.5%)	39(33.9%)	< 0.001
Hepatitis C	12(4.3%)	6(3.6%)	6(5.2%)	0.716
Alcohol	28(9.9%)	28(16.8%)	0	< 0.001
Autoimmune	27(9.6%)	6(3.6%)	21(18.3%)	< 0.001
Others	75(26.6%)	26(15.6%)	49(42.6%)	< 0.001
**Laboratory date**				
Hemoglobin (g/L)	85.29 ± 14.79	84.60 ± 14.33	86.28 ± 15.43	0.351
Platelet count (10^9^/L)	83.92 ± 55.03	78.19 ± 41.93	92.24 ± 69.19	0.035
creatinine (µmol/L)	65.35 ± 14.74	71.74 ± 13.77	56.07 ± 10.63	< 0.001
Total bilirubin (µmol/L)	19.19 ± 11.77	20.37 ± 12.68	17.48 ± 10.13	0.043
ALT (U/L)	26.70 ± 20.53	28.44 ± 21.78	24.16 ± 18.37	0.085
AST (U/L)	31.57 ± 17.84	32.20 ± 20.17	30.65 ± 13.81	0.474
Serum albumin (g/L)	36.13 ± 4.74	36.31 ± 4.72	35.88 ± 4.78	0.463
PT (s)	14.82 ± 1.98	15.25 ± 1.96	14.19 ± 1.84	< 0.001
INR	1.31 ± 0.18	1.36 ± 0.18	1.24 ± 0.16	< 0.001
D-dimer (g/L)	1.04 ± 1.26	1.09 ± 1.32	0.96 ± 1.15	0.396
Child-Pugh score	6.57 ± 1.49	6.71 ± 1.58	6.36 ± 1.35	0.050
MELD score	10.09 ± 2.39	10.60 ± 2.50	9.35 ± 1.99	< 0.001
Number of endoscopic treatments	2.62 ± 0.89	2.61 ± 0.88	2.63 ± 0.89	0.887
Number of bands	9.26 ± 3.09	9.79 ± 2.96	8.49 ± 3.11	< 0.001
PVT, n (%)	92(32.6%)	55(32.9%)	37(32.2%)	0.894
Ascites, n (%)	137(48.6%)	81(48.5%)	56(48.7%)	0.975
Diabetes mellitus, n (%)	61(21.6%)	38(22.8%)	23(20.0%)	0.581
NSBBs used	170(60.3%)	99(59.3%)	71(61.7%)	0.679
**Body Composition**				
BMI (kg/m^2^)	23.65 ± 3.09	24.01 ± 3.02	23.13 ± 3.13	0.019
SATI (cm/m^2^)	43.75 ± 24.83	36.85 ± 22.22	53.77 ± 25.08	< 0.001
VATI (cm/m^2^)	37.41 ± 20.69	37.24 ± 22.77	37.64 ± 17.31	0.867
TATI (cm/m^2^)	81.33 ± 41.17	74.39 ± 41.29	91.41 ± 39.01	0.001

Data are means ± standard deviations

Abbreviations: ALT, alanine aminotransferase; AST, aspartate aminotransferase; PT, prothrombin time; INR, international normalised ratio; MELD, Model For End Stage Liver Disease; PVT, portal vein thrombosis; NSBBs, nonselective beta-receptor blockers; BMI, body mass index; SATI, subcutaneous adipose tissue index; VATI, visceral adipose tissue index; TATI, total adipose tissue index

A median 31-month (IQR 13.8–40.0) follow-up period was conducted. 82 patients (29.1%) had rebleeding, and 29 patients (10.3%) passed away in follow-up period. Among the patients who rebleeded, 67 underwent additional endoscopic treatment, 9 were managed conservatively, 2 received transjugular intrahepatic portosystemic shunt (TIPS), and 4 experienced fatal outcomes due to uncontrolled bleeding. Among the 29 deceased patients, 4 and 18 succumbed due to uncontrolled bleeding and deteriorating liver function, respectively. Five patients died from hepatocellular carcinoma, and 2 deaths had unclear causes. Three patients underwent liver transplantation.

### Factors associated with rebleeding in cirrhotic patients

For assessing the relation of adipose tissue with rebleeding, a Cox proportional hazard regression was conducted. Univariate analysis identified several variables, including serum albumin, Child-Pugh score, MELD score, ascites, NSBB use, BMI, VATI, TATI, and SATI, to be correlated with rebleeding (Table 2). Upon multivariate regression, SATI, the continuous factor, significantly predicted rebleeding (HR 0.981, 95% CI, 0.971–0.991, *p* < 0.001) when additional predictors were adjusted (Table 2).

**Table 2:** rebleeding associated factors by univariate and multivariate COX regression analysis in cirrhotic patients

Characteristics	Univariate	*p*-value	Multivariate (SATI)	*p*-value	Multivariate (Low SATI)*	*p*-value
	HR (95%CI)		HR (95%CI)		HR (95%CI)	
Age	0.986(0.968–1.005)	0.149				
Gender	1.402(0.898–2.190)	0.137				
**Etiologic cause**						
Hepatitis B	1.177(0.769–1.803)	0.453				
Hepatitis C	1.878(0.818–4.315)	0.137				
Alcohol	1.083(0.543–2.161)	0.821				
Autoimmune	0.856(0.395–1.858)	0.695				
Others	0.694(0.412–1.168)	0.169				
**Laboratory date**						
Hemoglobin	0.991(0.977–1.005)	0.223				
Platelet count	0.999(0.995–1.003)	0.671				
Total bilirubin	1.014(0.996–1.031)	0.125				
Serum albumin	0.929(0.886–0.974)	0.002				
PT	1.051(0.939–1.175)	0.388				
Child-Pugh score	1.322(1.166-1.500)	< 0.001	1.288(1.127–1.473)	< 0.001	1.262(1.111–1.434)	< 0.001
MELD score	1.123(1.026–1.229)	0.012				
PVT	0.914(0.576–1.451)	0.704				
Ascites	2.169(1.397–3.367)	0.001				
NSBBs used	0.506(0.330–0.770)	0.002	0.610(0.396–0.939)	0.025	0.605(0.393–0.933)	0.023
**Body Composition**						
BMI	0.944(0.882–1.010)	0.093				
SATI	0.976(0.966–0.987)	< 0.001	0.981(0.971–0.991)	< 0.001		
VATI	0.986(0.975–0.998)	0.020				
TATI	0.989(0.983–0.995)	< 0.001				
Low SATI*	2.917(1.874–4.540)	< 0.001			2.511(1.604–3.932)	< 0.001

Abbreviations: CI, confidence interval; HR, hazard ratio; PT, prothrombin time; MELD, Model For End Stage Liver Disease; PVT, portal vein thrombosis; NSBBs, nonselective beta-receptor blockers; BMI, body mass index; SATI, subcutaneous adipose tissue index; VATI, visceral adipose tissue index; TATI, total adipose tissue index

*Defined as SATI < 30.15 cm2/m2 for male and < 39.82 cm2/m2 for female

According to X-tile software, we determined the best SATI thresholds as 30.15 cm^2^/m^2^ and 39.82 cm^2^/m^2^ for males and females, separately. Multivariate Cox analysis revealed that Child-Pugh score (HR 1.262, 95% CI, 1.111–1.434, *p* < 0.001), NSBB use (HR 0.605, 95% CI, 0.393–0.933, *p* = 0.023), and low SATI (male < 30.15 cm^2^/m^2^, female < 39.82 cm^2^/m^2^) (HR 2.511, 95% CI, 1.604–3.932, *p* < 0.001) independently determined the rebleeding incidence (Table 2).

### Cumulative rebleeding risk analysis

**Fig. 2:**
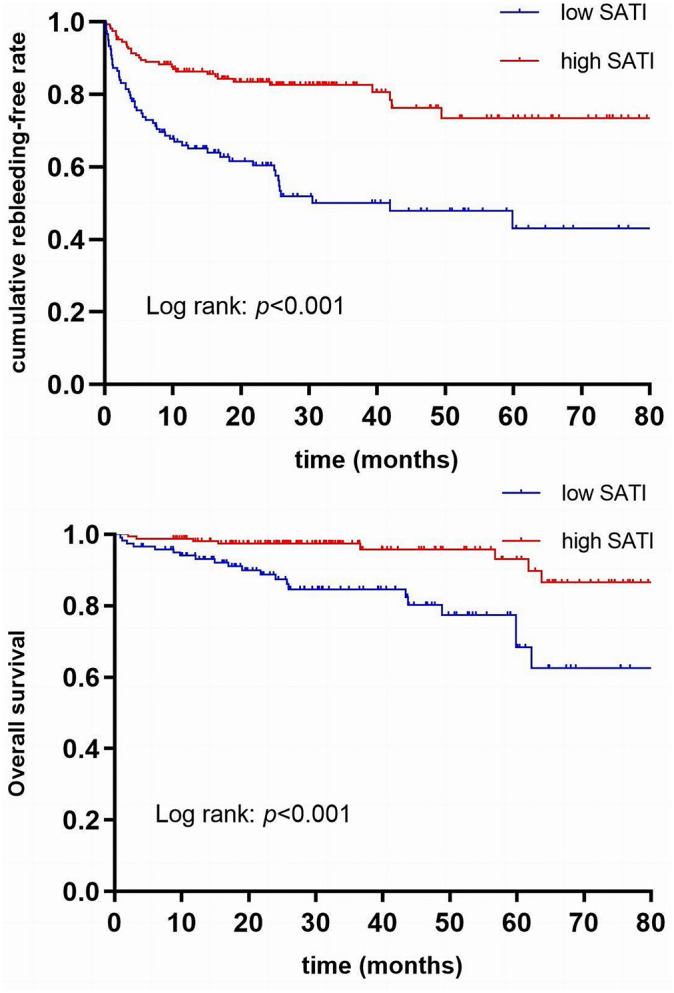
Kaplan–Meier curves of post-operative rebleeding and overall survival in high and low SATI group. (**A**) Higher cumulative incidence of rebleeding in patients with low SATI compared to the patients with high SATI (log-rank *p* < 0.001). (**B**) Higher cumulative incidence of mortality in patients with low SATI compared to the patients with high SATI (log-rank *p* < 0.001)

Based on the determined SATI thresholds of 30.15 cm/m2 for males and 39.82 cm/m2 for females, we divided patients as two groups: low SATI (119 patients, 42.2%) and high SATI (163 patients, 57.8%) groups. The high SATI group exhibited significantly higher BMI, VATI, TATI, Hb, and albumin levels, as well as reduced Child-Pugh scores, a lower proportion of male patients, and a decreased incidence of ascites in comparison with low SATI group (Table 3). Patients in low SATI group were related to an increased alcohol-induced cirrhosis risk. Kaplan-Meier analysis suggested that low SATI patients had a markedly elevated rebleeding risk compared to high SATI patients (*p* by log-rank test < 0.001; Fig. 2A).

**Table 3:** Clinical features of patients Associated with Low SATI

Characteristics	High SATI (n = 163)	Low SATI(n = 119)	*p*-value
Age (years)	55.17 ± 10.55	54.06 ± 11.06	0.392
Sex, male	86(52.8%)	81(68.1%)	0.010
**Etiologic cause, n (%)**			
Hepatitis B	81(49.7%)	59(49.6%)	0.985
Hepatitis C	6(3.7%)	6(5%)	0.576
Alcohol	11(6.7%)	17(14.3%)	0.037
Autoimmune	15(9.2%)	12(10.1%)	0.804
Others	50(30.7%)	25(21%)	0.070
**Laboratory date**			
Hemoglobin (g/L)	87.13 ± 15.29	82.76 ± 13.72	0.014
Platelet count (10^9^/L)	87.41 ± 57.40	79.14 ± 51.45	0.213
Creatinine (µmol/L)	64.59 ± 14.95	66.38 ± 14.45	0.318
Total bilirubin (µmol/L)	18.69 ± 11.48	19.87 ± 12.18	0.406
ALT (U/L)	25.88 ± 19.11	27.81 ± 22.37	0.438
AST (U/L)	30.60 ± 16.67	32.90 ± 19.32	0.286
Serum albumin (g/L)	36.67 ± 4.56	35.40 ± 4.90	0.026
PT (s)	14.66 ± 1.94	15.05 ± 2.01	0.104
INR	1.29 ± 0.18	1.33 ± 0.18	0.173
D-dimer (g/L)	1.07 ± 1.40	1.0 ± 1.02	0.695
Child-Pugh score	6.36 ± 1.42	6.86 ± 1.56	0.005
MELD score	9.90 ± 2.36	10.35 ± 2.41	0.118
Number of endoscopic treatments	2.61 ± 0.84	2.63 ± 0.95	0.831
Number of bands	9.15 ± 3.12	9.41 ± 3.04	0.478
PVT, n (%)	58(35.6%)	34(28.6%)	0.215
Ascites, n (%)	70(42.9%)	67(56.3%)	0.027
NSBB used, n (%)	110(67.5%)	60(50.4%)	0.004
**Body Composition**			
BMI (kg/m^2^)	25.03 ± 2.81	21.78 ± 8.84	< 0.001
VATI (cm^2^/m^2^)	48.42 ± 18.46	22.32 ± 12.42	< 0.001
TATI (cm^2^/m^2^)	108.19 ± 30.92	44.53 ± 18.99	< 0.001

Data are means ± standard deviations

Abbreviations: ALT, alanine aminotransferase; AST, aspartate aminotransferase; PT, prothrombin time; INR, international normalised ratio; MELD, Model For End Stage Liver Disease; PVT, portal vein thrombosis; NSBBs, nonselective beta-receptor blockers; BMI, body mass index; SATI, subcutaneous adipose tissue index; VATI, visceral adipose tissue index; TATI, total adipose tissue index

### Mortality-related factors among cirrhotic patients

Univariate analysis identified that several variables, including albumin, PT, Child-Pugh score, MELD score, BMI, VATI, TATI, and SATI, were related to mortality. Upon multivariate regression after adjusting for other mortality predictors, SATI remained to independently predict the mortality risk (HR 0.965, 95% CI, 0.944–0.986, *p* = 0.001; Table 4). When considering SATI as a binary variable, low Child-Pugh score (HR 1.383, 95% CI, 1.099–1.740, *p* = 0.006) and low SATI (HR 3.422, 95% CI, 1.489–7.864, *p* = 0.004) showed independent relation to increased mortality (Table 4). Furthermore, as revealed by Kaplan-Meier analysis, the mortality risk dramatically increased in low SATI group compared to high SATI group (*p* by log-rank test < 0.001; Fig. 2B).

**Table 4:** mortality associated factors by univariate and multivariate COX regression analysis in cirrhotic patients

	Univariate		Multivariate (SATI)		Multivariate (Low SATI)*	
Characteristics	HR (95%CI)	*p*-value	HR (95%CI)	*p*-value	HR (95%CI)	*p*-value
Age	1.205(0.989–1.063)	0.168				
Gender	1.935(0.857–4.372)	0.112				
**Etiologic cause**						
Hepatitis B	1.104(0.533–2.288)	0.790				
Hepatitis C	1.553(0.369–6.537)	0.548				
Alcohol	1.755(0.670–4.602)	0.253				
Autoimmune	0.457(0.062–3.386)	0.444				
Others	0.656(0.267–1.615)	0.359				
**Laboratory date**						
Hemoglobin	0.986(0.963–1.010)	0.259				
Platelet count	0.998(0.990–1.005)	0.585				
Total bilirubin	1.008(0.977–1.040)	0.625				
Serum albumin	0.891(0.820–0.968)	0.006				
PT	1.197(1.003–1.430)	0.047				
Child-Pugh score	1.493(1.195–1.864)	< 0.001	1.385(1.088–1.765)	0.008	1.383(1.099–1.740)	0.006
MELD score	1.173(1.014–1.358)	0.032				
PVT	0.941(0.428–2.066)	0.879				
Ascites	1.628(0.777–3.411)	0.196				
NSBB used	0.752(0.363–1.558)	0.443				
**Body Composition**						
BMI	0.856(0.757–0.967)	0.013				
SATI	0.958(0.937–0.980)	< 0.001	0.965(0.944–0.986)	0.001		
VATI	0.972(0.951–0.994)	0.011				
TATI	0.980(0.969–0.991)	< 0.001				
Low SATI*	4.179(1.846–9.459)	0.001			3.422(1.489–7.864)	0.004

Abbreviations: CI, confidence interval; HR, hazard ratio; PT, prothrombin time; MELD, Model For End Stage Liver Disease; PVT, portal vein thrombosis; NSBBs, nonselective beta-receptor blockers; BMI, body mass index; SATI, subcutaneous adipose tissue index; VATI, visceral adipose tissue index; TATI, total adipose tissue index

*Defined as SATI < 30.15 cm^2^/m^2^ for male and < 39.82 cm^2^/m^2^ for female

### Subgroup analysis based on NSBB usage

**Fig. 3:**
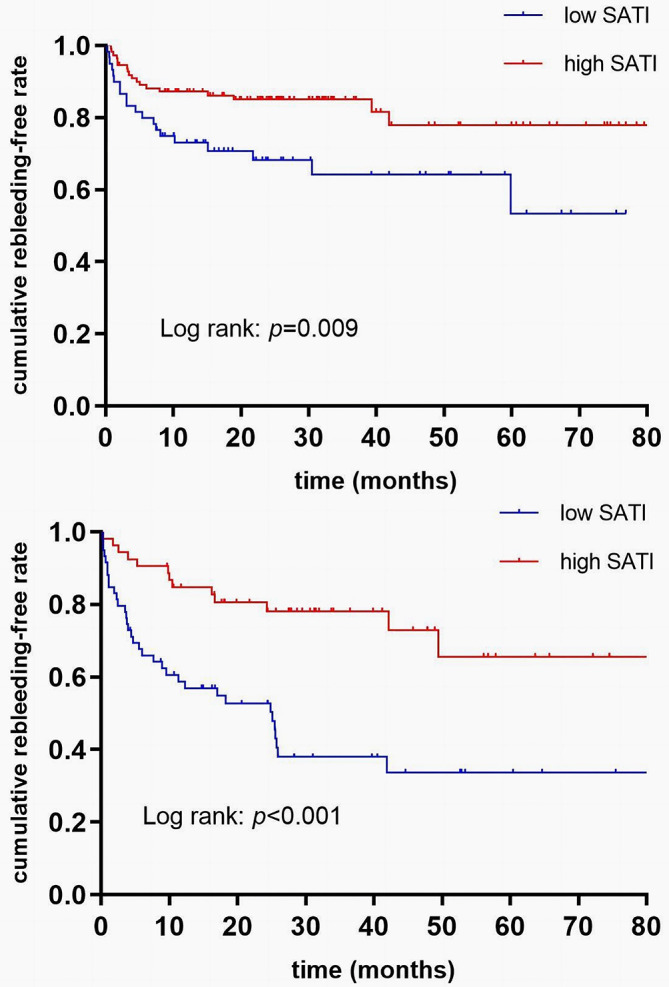
(**A**) Comparison of cumulative incidence of rebleeding between patients with low SATI and high SATI in group of standard therapy (log-rank p = 0.009). (**B**) Comparison of cumulative incidence of rebleeding between patients with low and high SATI in group of endoscopic treatment alone (log-rank *p* < 0.001)

Patients were categorized into two groups: those receiving NSBBs in addition to endoscopic therapy (n = 170) and those undergoing endoscopic therapy alone (n = 112). Upon multivariate regression after adjustment for additional predictors for rebleeding, low SATI remained a significant factor to predict rebleeding risk in standard therapy group (HR 2.020, 95% CI, 1.058–3.856, *p* = 0.033) and endoscopic therapy alone group (HR 3.027, 95% CI, 1.591–5.757, *p* = 0.001) (Table 5). According to Kaplan-Meier analysis, cumulative rebleeding risk was significantly different between high- and low-SATI groups in both standard therapy (*p* by log-rank test = 0.009; Fig. 3A) and the endoscopic therapy groups (*p* by log-rank test < 0.001; Fig. 3B).

**Table 5:** Subgroup analysis based on nonselective B-blockers usage

Variables	Univariate analysis HR (95%CI)	*p*-value	Multivariate analysis HR (95%CI)	*p*-value
**NSBBs use**				
Gender	1.563(0.798–3.060)	0.193		
Age	0.978(0.950–1.007)	0.139		
Serum albumin	0.901(0.834–0.973)	0.008		
Child-Pugh score	1.406(1.130–1.749)	0.002	1.352(1.088–1.681)	0.007
Ascites	2.323(1.199–4.503)	0.013		
BMI	0.958(0.865–1.061)	0.413		
VATI	0.990(0.974–1.007)	0.244		
Low SATI*	2.276(1.203–4.308)	0.011	2.020(1.058–3.856)	0.033
TATI	0.991(0.982–0.999)	0.025		
**NSBBs no used**				
Gender	1.203(0.663–2.182)	0.543		
Age	0.992(0.967–1.017)	0.520		
Serum albumin	0.948(0.894–1.005)	0.074		
Child-Pugh score	1.244(1.064–1.454)	0.006	1.220(1.035–1.438)	0.018
Ascites	1.925(1.067–3.470)	0.030		
BMI	0.949(0.867–1.038)	0.251		
Low SATI*	3.231(1.567–6.662)	0.001	3.027(1.591–5.757)	0.001
VATI	0.986(0.971–1.002)	0.089		
TATI	0.990(0.981–0.998)	0.013		

Abbreviations: CI, confidence interval; HR, hazard ratio; NSBBs, nonselective beta-receptor blockers; BMI, body mass index; VATI, visceral adipose tissue index; TATI, total adipose tissue index

*Defined as SATI < 30.15 cm^2^/m^2^ for male and < 39.82 cm^2^/m^2^ for female

## Discussion

The current study is the first to identify SATI as an essential factor to predict prognostic outcome in cirrhotic patients following endoscopic therapy. A low SATI is found to predict the occurrence of rebleeding and mortality. These findings emphasize the potential of SATI measurement for more accurately classifying risk in individual patients and identifying those who could benefit from nutritional interventions.

The relation of adipose tissue with survival of cirrhotic patients is always a hotspot research topic, yet no conclusive results are obtained. This is primarily because of the varying effects that different types of adipose tissues can have on the development of complications related to cirrhosis. Several studies have reported a correlation between reduced SATI among cirrhotic patients and the higher clinical decompensation and mortality risks, particularly among those awaiting liver transplantation [14, 15]. However, Kimura et al. recently found that the high VATI, as opposed to SATI, exhibited a significant predictive value for mortality in cirrhotic patients by impairing liver function [16]. Notably, high VATI did not show a significant difference. The distribution of VAT accumulation is primarily observed in patients with alcohol-related or metabolic syndrome-related cirrhosis, negatively impacting their prognosis [11, 17]. In this study, 49.6% of the individuals were found to have cirrhosis related to Hepatitis B Virus (HBV) infection, whereas only 9.9% had alcohol-related cirrhosis. This difference in etiologies may explain why only SATI was significantly associated with rebleeding and mortality risks among patients with cirrhosis.

Precise mechanisms underlying how low SATI negatively affects the prognosis of cirrhosis patients remain incompletely understood. It has been postulated that SAT primarily secretes anti-inflammatory cytokines, such as adiponectin, which possess the potential to alleviate hepatic inflammation and fibrogenesis [5, 18]. Notably, Rodrigues et al. reported that SATI significantly decreased in high hepatic venous pressure gradient (HVPG) group relative to low HVPG group [6]. HVPG is widely recognized as the most precise prognostic indicator for clinical decompensation and poor prognosis of patients with cirrhosis [19]. Hence, the depletion of SAT may be linked to chronic inflammation and an increase in portal pressure, thereby deteriorating prognosis of these patients. Additionally, subcutaneous fat has an important effect on energy and lipid storage, which exerts beneficial effects on metabolism by modulating insulin sensitivity, as well as lipid and glucose metabolism [20, 21]. In mouse models, the depletion of SAT has been associated with increased insulin resistance, elevated levels of TNF-α, and induced metabolic deterioration [22]. Therefore, low SATI may serve as an indicator of significant energy depletion caused by cirrhosis, resulting in unfavorable clinical outcomes such as rebleeding and mortality.

Patients with cirrhosis frequently experience inadequate dietary intake, which can be attributed to abdominal distention resulting from ascites and impaired nutrient absorption. Consequently, cirrhotic patients are susceptible to malnutrition, a factor that further compromises their survival and hastens the progression of decompensation [23, 24]. Additionally, it is worth noting that SATI is notably lower in malnourished cirrhotic patients, and a low SATI may serve as an indicator for protein-energy malnutrition and energy reserve deficiency [25]. This underscores the close relationship between SATI and nutritional states in these patients. Furthermore, dietary restriction represents the fundamental aspect of endoscopic therapy, which can exacerbate the issue of malnutrition among cirrhotic patients. By assessing SATI, healthcare providers can better estimate the malnutrition risk among cirrhotic patients, thereby identifying those who may benefit from nutritional intervention both before and after undergoing endoscopic therapy, ultimately leading to improved patient outcomes. According to prior results, early oral nutrition is safe and beneficial for patient recovery following endoscopic treatment [26]. Additionally, a randomized controlled trial has indicated the advantages of supplementation, including branched-chain amino acids and nutritional energy supplements, in cirrhotic patients undergoing endoscopic therapy [27]. It is essential to conduct further prospective investigations to explore whether enhancing the nutritional status of cirrhotic patients with low SATI can contribute to improved prognosis.

Numerous studies have consistently highlighted the pivotal role of NSBB therapy in secondary prophylaxis, with patients receiving endoscopic therapy alone displaying a heightened risk of recurrent bleeding and mortality when compared to those receiving NSBBs in combination with endoscopic treatment [28, 29]. This study similarly revealed a reduced rate of rebleeding among patients who incorporated NSBBs into their treatment regimen when compared to those who did not. However, it is worth acknowledging the real-world challenges in achieving standardized treatment for all patients due to various factors, including intolerance or contraindications to NSBBs, poor patient compliance, and other logistical hurdles. Therefore, a subgroup analysis based on the use of NSBBs was performed, and the results were consistent with the overall findings, underscoring the critical role of quantitative SATI assessment in evaluating the risk of rebleeding among patients, irrespective of whether they received NSBB therapy or not. In contrast, NSBB therapy was not found to be significantly related to the risk of mortality in the present study. This observation may be caused by the majority of patients in this cohort falling under Child-Pugh class A. In alignment with existing systematic reviews and meta-analyses, NSBBs have been shown to efficiently decrease rebleeding incidence among patients classified as Child-Pugh class A, with no discernible impact on overall survival [30].

## Study strengths and limitations

The strengths in the present work are outlined below. First, it marked the first instance of reporting the predictive role of SATI in assessing the prognosis after endoscopic treatment. This introduction of a new metric for prognostic evaluation suggests that SATI integration into a comprehensive model may enhance the accuracy of prognosis prediction for patients undergoing endoscopic treatment. Second, the ability of SATI to predict the nutritional status underlines the importance of maintaining favorable nutritional conditions after endoscopic treatment. This also underscores the significance of early nutritional screening and essential nutritional support, particularly for patients with low SATI levels. Last, considering the widespread utilization of CT scans in clinical practice, it presents a swift and precise method to quantify adipose tissue when assessing patients’ nutritional status. Consequently, incorporating this assessment into regular patient follow-ups allows for the customization of strategies to improve patient prognosis after endoscopic treatment.

This study does have certain limitations. Possible selection bias is inevitable due to its retrospective nature. Additionally, the insufficient sample size and low incidence of death events may limit the statistical power to comprehensively analyze the relationship between SATI and mortality among cirrhotic patients after endoscopic therapy. Lastly, the absence of portal pressure measurements among the patients necessitates further exploration of the relationship between SATI and portal hypertension in larger prospective cohort studies.

## Conclusion

In conclusion, low SATI appears to indicate insufficient energy reserves and protein-energy malnutrition, correlating with elevated risks of rebleeding and mortality among cirrhotic patients who had received endoscopic treatment. The measurement of SATI has the potential to provide a more precise risk classification for cirrhotic patients and identify individuals who may benefit from nutritional interventions.

## Data Availability

The datasets used and/or analyzed during the current study are available from the corresponding author on reasonable request.

## Abbreviations

ALT: alanine aminotransferase
AST: aspartate aminotransferase
BMI: body mass index
CI: confidence interval
CT: computed tomography
EGVB: esophagogastric variceal bleeding
EVL: endoscopic variceal ligation
Hb: hemoglobin
HBV: Hepatitis B Virus
HU: Hounsfield Unit
HR: hazard ratio
HVPG: high hepatic venous pressure gradient
INR: international normalized ratio
IQR: interquartile ranges
MELD: Model for End-Stage Liver Disease
NAFLD: non-alcoholic fatty liver disease
NSBBs: Nonselective β-blockers
PT: prothrombin time
PVT: portal vein thrombosis
SAT: subcutaneous adipose tissue
SATI: subcutaneous adipose tissue Index
SD: standard deviation
TATI: total adipose tissue index
TIPS: transjugular intrahepatic portosystemic shunt
VAT: visceral adipose tissue
VATI: visceral adipose tissue index
